# Metabolic Adaptations During *Staphylococcus aureus* and *Candida albicans* Co-Infection

**DOI:** 10.3389/fimmu.2021.797550

**Published:** 2021-12-08

**Authors:** Kara R. Eichelberger, James E. Cassat

**Affiliations:** ^1^ Department of Pediatrics, Division of Pediatric Infectious Diseases, Vanderbilt University Medical Center, Nashville, TN, United States; ^2^ Department of Pathology, Microbiology, and Immunology, Vanderbilt University Medical Center, Nashville, TN, United States; ^3^ Vanderbilt Center for Bone Biology, Vanderbilt University Medical Center, Nashville, TN, United States; ^4^ Department of Biomedical Engineering, Vanderbilt University, Nashville, TN, United States; ^5^ Vanderbilt Institute for Infection, Immunology, and Inflammation (VI4), Vanderbilt University Medical Center, Nashville, TN, United States

**Keywords:** *Candida albicans*, *Staphylococcus aureus*, polymicrobial infection, polymicrobial biofilm, fungal-bacterial interactions

## Abstract

Successful pathogens require metabolic flexibility to adapt to diverse host niches. The presence of co-infecting or commensal microorganisms at a given infection site can further influence the metabolic processes required for a pathogen to cause disease. The Gram-positive bacterium *Staphylococcus aureus* and the polymorphic fungus *Candida albicans* are microorganisms that asymptomatically colonize healthy individuals but can also cause superficial infections or severe invasive disease. Due to many shared host niches, *S. aureus* and *C. albicans* are frequently co-isolated from mixed fungal-bacterial infections. *S. aureus* and *C. albicans* co-infection alters microbial metabolism relative to infection with either organism alone. Metabolic changes during co-infection regulate virulence, such as enhancing toxin production in *S. aureus* or contributing to morphogenesis and cell wall remodeling in *C. albicans*. *C. albicans* and *S. aureus* also form polymicrobial biofilms, which have greater biomass and reduced susceptibility to antimicrobials relative to mono-microbial biofilms. The *S. aureus* and *C. albicans* metabolic programs induced during co-infection impact interactions with host immune cells, resulting in greater microbial survival and immune evasion. Conversely, innate immune cell sensing of *S. aureus* and *C. albicans* triggers metabolic changes in the host cells that result in an altered immune response to secondary infections. In this review article, we discuss the metabolic programs that govern host-pathogen interactions during *S. aureus* and *C. albicans* co-infection. Understanding *C. albicans-S. aureus* interactions may highlight more general principles of how polymicrobial interactions, particularly fungal-bacterial interactions, shape the outcome of infectious disease. We focus on how co-infection alters microbial metabolism to enhance virulence and how infection-induced changes to host cell metabolism can impact a secondary infection.

## Introduction

Microorganisms co-exist as polymicrobial communities in the human body, sharing colonization niches and competing for resources. Interactions among these commensal microbial communities facilitate persistence of microorganisms that promote health. However, in the context of a polymicrobial infection, interactions among co-infecting pathogens may exacerbate disease. Polymicrobial infections can develop when infection by one organism creates a favorable host niche for colonization by additional organisms, when two or more organisms are simultaneously introduced into the body, or when host immune defenses are weakened (1). For example, polymicrobial infections frequently develop in individuals with compromised host immune responses or disrupted biological barrier function, in which there may exist a more favorable environment for co-infection (2–4). Co-infecting pathogens can behave antagonistically, additively, or synergistically to alter disease outcome relative to mono-microbial infections (1). However, many polymicrobial infections are associated with more severe disease relative to mono-microbial infections (5–7). Because polymicrobial infections can alter the outcome of disease, it is critical to understand how the presence of co-infecting pathogens affect microbial physiology and subsequent host responses during infection.

Bacteria and fungi are frequently co-isolated from commensal microbial communities, and inter-species cross-talk facilitates stable and asymptomatic colonization of these organisms (8). However, cross-talk between bacteria and fungi can also contribute to disease progression in the context of a polymicrobial infection. The opportunistic fungal pathogen *Candida albicans* and the Gram-positive bacterium *Staphylococcus aureus* are two of the most commonly co-isolated pathogens from mixed fungal-bacterial infections in the bloodstream as well as from biofilm-associated diseases such as cystic fibrosis, periodontitis, and catheter-associated infections (4, 8–10). The frequency of mixed *C. albicans-S. aureus* infections is in part due to shared colonization sites in the human body as well as a propensity for these microorganisms to interact and form polymicrobial biofilms (8, 10, 11). *C. albicans* and *S. aureus* both asymptomatically colonize the skin or gastrointestinal tract, yet under certain circumstances they can also cause severe invasive disease (12, 13). A key feature of the virulence of both *C. albicans* and *S. aureus* is the ability to utilize a variety of nutrient sources to establish infection in diverse host niches (14, 15). *C. albicans* virulence is mediated in large part by a morphologic switch from growth as budding yeast to filamentous hyphae. *C. albicans* yeast colonize skin and the gastrointestinal mucosa (12). During invasive infection, *C. albicans* hyphal growth penetrates epithelial layers and causes tissue damage, while *C. albicans* yeast disseminate through the bloodstream and colonize other organs (12). *C. albicans* mutants that are genetically locked in either morphology are attenuated during infection, highlighting the importance of the ability to switch between the yeast and the hyphal forms to cause disease (12, 16). However, recent studies have challenged this dogma, demonstrating that certain *C. albicans* yeast-locked strains retain virulence in disseminated infection due to the metabolic advantages of yeast growth over hyphal growth (17). *S. aureus* virulence is driven by a variety of mechanisms to combat host responses and adapt to host environments. This includes production of toxins and immunomodulatory proteins that evade host immune responses (18–21).

*C. albicans-S. aureus* co-infection worsens invasive disease relative to infection with either organism alone. Some of the earliest studies investigating *C. albicans-S. aureus* co-infection identified that this polymicrobial interaction is associated with greater mortality when both organisms are inoculated into the peritoneal cavity of mice simultaneously (22–24). The enhanced virulence of *C. albicans-S. aureus* co-infection is due in part to physical and chemical interactions between the two microorganisms that influence microbial metabolism, virulence, and physiology. Host immune responses also play an important role in contributing to the enhanced virulence of *C. albicans-S. aureus* co-infection. Co-infection skews the balance of pro-inflammatory and anti-inflammatory cytokine production towards greater inflammation. Altered host responses can occur through direct interactions of host cells with the microbes that facilitate microbial dissemination, as well as through increased pro-inflammatory cytokine production by host cells as a response to co-infection (25, 26). Paradoxically, outcomes of invasive *S. aureus* infections are improved when *S. aureus* is inoculated after a *C. albicans* infection, suggesting cross-species protection in the context of secondary infection (27). In this review, we discuss advances towards understanding the factors that contribute to the changes in disease progression during *C. albicans-S. aureus* co-infection, with a focus on interactions that worsen disease outcomes. We highlight the mechanisms by which virulence is altered during acute polymicrobial infection, as well as how interactions during polymicrobial biofilm growth influence virulence and antimicrobial resistance. Finally, we discuss how polymicrobial infection alters host cell responses during co-infection and how sequential infection is protective rather than deleterious.

## *C. albicans* Influence on *S. aureus* Virulence

**Figure 1:**
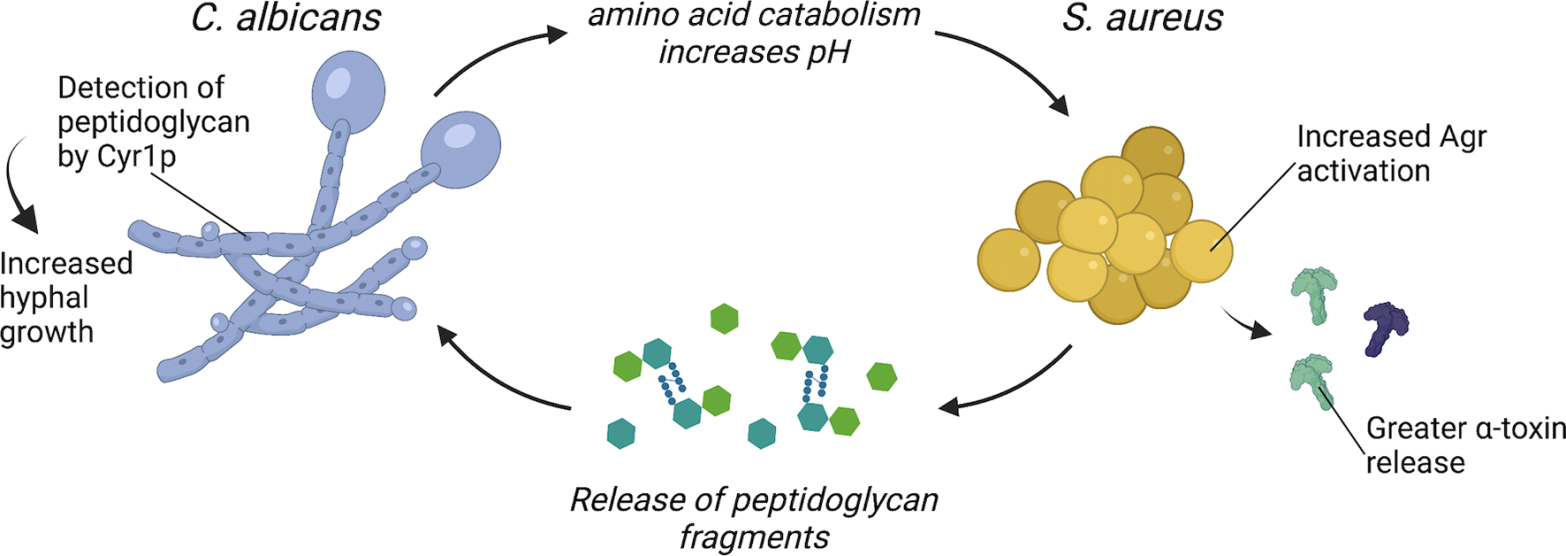
*C. albicans* and *S. aureus* interact to enhance virulence. *C. albicans* enhances *S. aureus* Agr activation through amino acid metabolism-driven pH modification and morphogenesis signaling. *S. aureus* may enhance *C. albicans* virulence through the release of peptidoglycan fragments that are sensed by Cyr1p and trigger hyphal morphogenesis.

Co-inoculation of *C. albicans* and *S. aureus* into the peritoneal cavity of mice results in 100% mortality, while inoculation of the same dose of either organism alone does not result in lethal disease (25). Recent work identified that *C. albicans* influences *S. aureus* quorum sensing to augment its virulence, which contributes to the lethality of polymicrobial intra-peritoneal infection (**Figure 1**). Co-culture of *C. albicans* with *S. aureus* enhances the *S. aureus* quorum-sensing system Agr, or accessory gene regulator (28). The Agr system regulates a variety of toxins that are important for *S. aureus* pathogenesis, including α-toxin (21, 29). Todd et al. determined that co-culture of *C. albicans* with *S. aureus* enhances α-toxin levels *in vitro*, and co-inoculation of *C. albicans* and *S. aureus* into the peritoneum also resulted in greater α-toxin levels in the peritoneal lavage fluid relative to *S. aureus* mono-infection (28). While antibody treatment that neutralizes α-toxin in mice partially alleviated the mortality in a *C. albicans-S. aureus* co-infection, injection of purified α-toxin combined with live *C. albicans* was not sufficient to induce enhanced mortality (28). Thus, lethal synergism in this model may require either live organisms or involve additional *S. aureus* virulence factors.

One mechanism proposed for enhanced activation of *S. aureus* Agr system during co-culture with *C. albicans* requires *C. albicans* amino acid metabolism. During infection, *C. albicans* can catabolize amino acids and utilize these nutrients as a carbon source (14). Amino acids are imported *via* amino acid permeases, which are regulated by the transcription factors Stp1p and Stp2p. As *C. albicans* catabolizes amino acids, it exports ammonia, which alkalinizes the extracellular media and promotes *C. albicans* hyphal formation (30). *C. albicans stp2*Δ/Δ is defective in the alkalinization of the extracellular environment and is more efficiently killed during macrophage infection (30). The Agr system is sensitive to pH and optimal activity occurs at neutral pH (31–33). Therefore, it was hypothesized that *C. albicans* alkalinization of the extracellular media as a byproduct of amino acid metabolism provides an optimal pH for activation of the *S. aureus* Agr system during co-culture (34). Indeed, *S. aureus* co-cultured with *C. albicans stp2*Δ/Δ produces less α-toxin relative to *S. aureus* co-cultured with wild-type *C. albicans* (34). However, the role of *C. albicans* amino acid catabolism in promoting enhanced virulence during co-infection with *S. aureus in vivo* remains to be determined.

An additional mechanism by which *C. albicans* may enhance *S. aureus* virulence during co-infection requires *C. albicans* morphogenesis signaling. Mice survive intraperitoneal co-infection of *S. aureus* and the *C. albicans* morphogenesis mutant *efg1*Δ/Δ, while mice inoculated with *S. aureus* and wild-type *C. albicans* all succumb to disease (35). Efg1p is a transcriptional regulator that induces hyphal gene expression and filamentous growth, and an *EFG1* mutant, which can only grow as yeast, is highly attenuated *in vivo* (36, 37). To test if enhanced mortality during intraperitoneal co-infection requires a specific *C. albicans* morphology, Nash et al. inoculated mice with *S. aureus* and either a *C. albicans* yeast-locked strain or a *C. albicans* hyphal-locked strain (38). However, *S. aureus* co-inoculated with either yeast-locked *C. albicans* or hyphal-locked *C. albicans* is as lethal as co-infection with wild-type *C. albicans* in this model (38). Considering that morphology-locked strains are typically attenuated *in vivo*, it is surprising that morphology-locked *C. albicans* strains can still enhance *S. aureus* virulence during intraperitoneal co-infection (16, 36). There may be additional morphology-independent processes regulated by Efg1p that contribute to lethal synergism during polymicrobial intra-abdominal infection. In addition to inducing filamentous growth in *C. albicans*, Efg1p is a master regulator of metabolic genes, as well as genes involved in adhesion (39). For example, *C. albicans efg1*Δ/Δ has significantly reduced transcript levels for almost all glycolytic genes and several tricarboxylic acid cycle genes (40). Thus, *C. albicans* metabolism, as regulated by Efg1p, may play an additional role in enhancing *S. aureus* virulence during intra-abdominal polymicrobial infection.

## *S. aureus* Influence on *C. albicans* Virulence

The mechanisms by which *S. aureus* directly promotes *C. albicans* virulence are less clear. However, one bacterial-derived molecule with potent effects on *C. albicans* morphology and metabolism is peptidoglycan. Serum is a potent inducer of *C. albicans* hyphal formation, and an analysis of the hyphal-stimulating fractions of human and bovine serum found structures resembling bacterial peptidoglycan fragments (37, 41, 42). Partial hydrolysis of peptidoglycan purified from the Gram-positive bacterium *S. aureus* or the Gram-negative bacterium *Escherichia coli* potently induces hyphal formation (41). These peptidoglycan fragments are imported by *C. albicans* and sensed by the adenylyl cyclase protein Cyr1, which activates the Protein Kinase A (PKA) pathway and induces hyphal gene expression (41). One hypothesis is that the source of peptidoglycan fragments present in serum is from turnover of the bacteria that comprise the intestinal microbiome (42). Tan et al. confirmed that culturing *C. albicans* adjacent to both Gram-positive and Gram-negative bacteria induced hyphal formation, and this effect could be further augmented by treatment with β-lactam antibiotics (43). β-lactams disrupt crosslinking of new peptidoglycan fragments, freeing peptidoglycan molecules from the bacterial cell wall during the process of killing the bacteria (44). Mice that were treated with β-lactam antibiotics before and after *C. albicans* oral inoculation developed greater *C. albicans* hyphal formation in the gut (43). The β-lactam-treated mice also had higher *C. albicans* burdens in the kidneys, indicating increased dissemination to peripheral organs following β-lactam treatment (43). However, signaling among the various bacterial organisms that comprise the intestinal microbiota and *C. albicans* likely also influence *C. albicans* morphology and pathogenesis in the gastrointestinal tract.

*N*-Acetylglucosamine (GlcNAc) is also a component of peptidoglycan that can influence *C. albicans* metabolism and virulence. During infection, *C. albicans* can metabolize alternative carbon sources, such as GlcNAc, to facilitate host adaptation and survival (14). GlcNAc is also found as a component of chitin in fungal cell walls and as a part of the extracellular matrix in animals (45). However, bacteria release GlcNAc during cell wall remodeling and peptidoglycan turnover and may provide a significant source of GlcNAc to *C. albicans* cells during mixed fungal-bacterial infections (45, 46). GlcNAc is imported into the fungal cell *via* Ngt1p, is phosphorylated, and either enters an anabolic pathway for the synthesis of chitin or is catabolized for use in glycolysis (45, 47, 48). Interestingly, *C. albicans* mutants that cannot phosphorylate and metabolize imported GlcNAc still form hyphal filaments following GlcNAc exposure (49). This indicates a role for GlcNAc as a signaling molecule in *C. albicans* that is independent of its role as a nutrient and carbon source. However, metabolism of GlcNAc can also induce hyphal formation, as it releases ammonia and alkalinizes the extracellular media, which triggers hyphal formation (50). GlcNAc metabolism is important for alkalinization of the macrophage phagosome following phagocytosis of *C. albicans* yeast, which promotes *C. albicans* hyphal growth and survival within the macrophage (51). These studies collectively demonstrate that components of bacterial peptidoglycan can have potent effects on *C. albicans* metabolism, morphogenesis, and subsequent virulence.

During co-infection, *S. aureus* grows in close association with *C. albicans* hyphae (24). Since *S. aureus* releases peptidoglycan fragments during growth (52), it is feasible that peptidoglycan recycling and remodeling plays a role in altering *C. albicans* metabolism and morphogenesis through a ready supply of released peptidoglycan fragments (**Figure 1**). Peptidoglycan detection by *C. albicans* could be an additional mechanism contributing to the lethal synergism exhibited by *C. albicans* and *S. aureus* co-infections. Whether the ability of *C. albicans* to sense and respond to peptidoglycan released by neighboring bacteria is also important for *C. albicans* existence as a commensal within the gastrointestinal tract remains to be determined.

## *C. albicans-S. aureus* Biofilm Interactions

**Figure 2:**
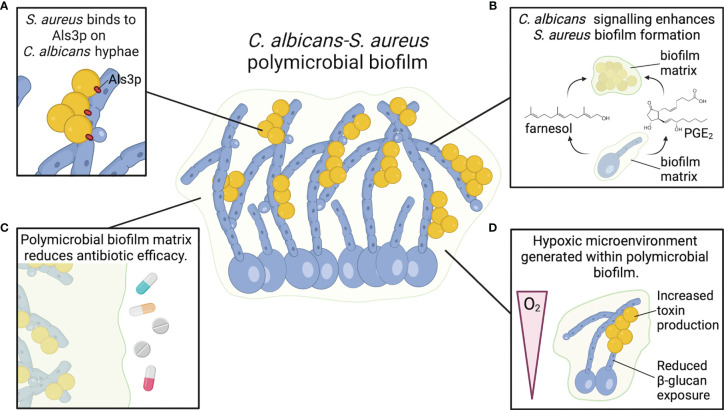
*C. albicans* and *S. aureus* form robust polymicrobial biofilms. **(A)** *S. aureus* adheres to Als3 protein on *C. albicans* hyphae, promoting strong attachment and enhancing biofilm growth. **(B)** *C. albicans* chemical signals farnesol and PGE_2_ enhance *S. aureus* biofilm growth and biomass. **(C)** Biofilm matrix components, particularly β-glucan, act as a physical barrier to resist antimicrobials and promote *S. aureus* survival. **(D)** *C. albicans* biofilms generate a hypoxic microenvironment that may enhance *S. aureus* toxin production.

Biofilms pose a major clinical problem as they display tolerance to standard antimicrobial treatments and can become a niche for persistent infections. In addition to their ability to form robust biofilms during mono-infection, *C. albicans* and *S. aureus* have a propensity to form robust polymicrobial biofilms during co-infection (4, 53, 54). These are often associated with indwelling medical devices, such as catheters (55). However, certain disease conditions, such as within the cystic fibrosis lung, are also a niche for polymicrobial biofilm growth of *C. albicans* and *S. aureus* (5). In this section, we will review metabolic adaptations of both organisms during polymicrobial biofilm growth. We discuss how polymicrobial biofilm growth contributes to the altered virulence observed during a *C. albicans-S. aureus* co-infection relative to infection with either organism alone (**Figure 2**).

### Polymicrobial Biofilm Structure and Metabolism

*C. albicans* and *S. aureus* are tightly associated in polymicrobial biofilms. *S. aureus* forms microcolonies within the *C. albicans* biofilm matrix, and the bacteria primarily attach to the hyphal form of *C. albicans* (**Figure 2A**) (56–58). Binding to *C. albicans* hyphae is predominantly mediated by the *C. albicans* hyphal-specific protein Als3, which functions as an adhesin (59). However, in the presence of serum, *S. aureus* adheres to *C. albicans als3*Δ/Δ filaments to the same degree as wild-type filaments, indicating that other factors may play a role in adherence of *S. aureus* to *C. albicans* hyphae under different conditions (60). In addition to strong adherence to hyphal filaments, *S. aureus* colony forming units (CFU) are also significantly higher when grown with *C. albicans* in a polymicrobial biofilm relative to mono-microbial biofilm (56, 61, 62). However, *C. albicans* CFU remain the same whether grown in biofilms alone or with *S. aureus* (56). Biofilm growth is reduced when *S. aureus* is co-cultured with *efg1*Δ/Δ *cph1*Δ/Δ and *bcr1*Δ/Δ *C. albicans* mutants, but these mutants are also unable to form robust biofilms during *C. albicans* mono-microbial growth (60). Interestingly, *S. aureus* biofilm growth is enhanced when co-cultured with amphotericin-B killed, but not formalin killed, *C. albicans* biofilm (60). Amphotericin-B disrupts fungal cell membranes to kill *C. albicans*, while formalin kills cells by cross-linking proteins. Therefore, enhanced *S. aureus* biofilm growth during co-culture with *C. albicans* is not dependent on a process mediated by live *C. albicans* cells, but rather a surface protein that can be neutralized by formalin. Fungal glucans are an additional *C. albicans* cell wall component present in the extracellular matrix material of *C. albicans* biofilms, and glucans enhance *S. aureus* biofilm formation when supplied exogenously (63).

The differences in *C. albicans* and *S. aureus* behavior and physiology within a polymicrobial biofilm may be partly mediated by metabolic changes. Proteomic analysis of *C. albicans-S. aureus* polymicrobial biofilm growth identified an increased abundance of proteins primarily involved in metabolism and stress response for both organisms relative to mono-microbial biofilms (57). Metabolic exchange within the polymicrobial biofilm could also influence biofilm formation and biomass. For example, glucose is the most abundant monosaccharide present in the extracellular matrix of a *C. albicans* mono-culture biofilm (64). Because glucose enhances *S. aureus* biofilm production, greater availability of glucose provided by the *C. albicans* biofilm matrix may also enhance *S. aureus* growth and biofilm production, contributing to greater biomass in the polymicrobial biofilms (65). Altered metabolism in *C. albicans-S. aureus* biofilm may also contribute to enhanced persistence of the organisms *in vivo*. *S. aureus* biofilms produce lactate that modulates host macrophages and myeloid-derived suppressor cells (MDSC) to produce more IL-10, which has an anti-inflammatory effect that enables persistence of *S. aureus* biofilms *in vivo* (66). *S. aureus-*produced lactate may also have effects on *C. albicans* cells within the polymicrobial biofilm. Lactate induces reorganization of the fungal cell wall to reduce, or mask, exposure of β-glucan on *C. albicans* hyphae (67). Because β-glucan is a potent immunostimulatory factor, the response to lactate reduces immune detection, allowing greater *C. albicans* persistence and survival during infection. While most studies have focused on synergistic metabolic interactions occurring within polymicrobial biofilms, broad metabolomic analyses to characterize both synergistic and antagonistic interactions between *C. albicans* and *S. aureus* are needed.

### Chemical Signaling in *C. albicans-S. aureus* Biofilms

Several chemicals produced by both *S. aureus* and *C. albicans* can influence polymicrobial biofilm formation through cross-species signaling. One of the best-studied chemical signals that can alter both *C. albicans* and *S. aureus* physiology is farnesol (68). Farnesol is a quorum-sensing molecule derived from glycolytic products *via* the sterol pathway in *C. albicans* (68). Farnesol blocks the *C. albicans* morphological transition from yeast to hyphae under high cell density (69). As *C. albicans* hyphal growth is required for the development of a biofilm, farnesol inhibits *C. albicans* biofilm formation (70). Depending on the concentration, farnesol exerts both synergistic and antagonistic effects on *S. aureus* physiology (4). High concentrations of farnesol were first determined to inhibit *S. aureus* biofilm growth, induce cell death, and increase sensitivity of a methicillin-sensitive *S. aureus* strain towards a variety of different classes of antibiotics (71). Farnesol can also inhibit *S. aureus* lipases, which hydrolyze host lipids to free fatty acids that promote colonization (72). However, low levels of farnesol promote biofilm formation in *S. aureus* (62). Additionally, Kong et al. determined that low concentrations of farnesol could increase *S. aureus* resistance to antibiotics *via* a cellular stress response (73). RNAseq analysis of *S. aureus* exposed to farnesol revealed an increase in expression of genes involved in stress response as well as protection against oxidative stress (74). Accordingly, farnesol treatment induces greater *S. aureus* resistance to killing by H_2_O_2_ (74). The ultimate effects of farnesol on *S. aureus* during infection may vary spatially or temporally within the polymicrobial biofilm based on the changes in *C. albicans* production of farnesol during development of the polymicrobial biofilm.

An additional chemical signaling molecule involved in *C. albicans* biofilm formation is prostaglandin E2 (PGE_2_). *C. albicans*, in addition to other pathogenic fungi, can synthesize prostaglandins *de novo* or following supplementation with arachidonic acid, which is metabolized to produce prostaglandins *via* cyclooxygenase (COX) enzymes (75). The prostaglandins synthesized by *C. albicans* stimulate germ tube formation, which is the precursory step to hyphal growth (75). Furthermore, *C. albicans* biofilm formation is significantly reduced following the use of COX inhibitor drugs to block prostaglandin biosynthesis (76). PGE_2_ added to *S. aureus* mono-culture stimulated greater biofilm growth relative to untreated *S. aureus* biofilms, mimicking the enhanced biomass following co-culture with *C. albicans* (62). These data suggest that PGE_2_ may be another fungal-derived molecule that influences biofilm growth of both organisms in a polymicrobial biofilm (**Figure 2B**).

The effects of *S. aureus* chemical signals on *C. albicans* biofilm formation and physiology are less understood. When *S. aureus* and *C. albicans* are added together to form a polymicrobial biofilm, *S. aureus* increases attachment of *C. albicans* to plastic surfaces (77). Additionally, conditioned media from a mature *S. aureus* biofilm significantly enhances *C. albicans* biofilm formation, suggesting *S. aureus* secreted factors contribute to biofilm formation (77). The *S. aureus* factor(s) that enhances *C. albicans* biofilm remains unknown. As discussed earlier, peptidoglycan fragments induce *C. albicans* hyphal formation (41, 43). Because hyphal formation is a requisite step in biofilm formation, it is possible that peptidoglycan fragments present in the *S. aureus* conditioned media are enhancing *C. albicans* biofilm. *S. aureus* can also negatively regulate biofilm formation through the production of staphylokinase (Sak), which reduces biomass and metabolic activity of *C. albicans-S. aureus* polymicrobial biofilm by causing detachment of cells from the biofilm (78). Sak also reduced *C. albicans* gene expression of morphogenesis regulators (78). However, it is unclear if Sak-mediated changes in *C. albicans* gene expression are directly mediated by Sak or if it is an indirect effect resulting from detachment of *C. albicans* and *S. aureus* cells from the polymicrobial biofilm. The effects of *S. aureus* chemicals and other secreted factors on polymicrobial biofilm formation during infection require further investigation.

### Antimicrobial Resistance in *C. albicans-S. aureus* Biofilms

Biofilm growth reduces antibiotic efficacy towards multiple organisms, including *S. aureus* and *C. albicans*, presenting a major barrier for treating infection. In addition to enhanced growth and biomass of a polymicrobial biofilm, *C. albicans-S. aureus* biofilms exhibit greater resistance to killing by antimicrobials relative to mono-microbial biofilms (**Figure 2C**) (56). Vancomycin is less effective at killing *S. aureus* when *S. aureus* is grown in a biofilm with *C. albicans*, but there are no changes in amphotericin-B fungicidal activity towards *C. albicans* (56). Decreased antibiotic efficacy was also observed when mice were surgically implanted with small catheters coated in *S. aureus* and *C. albicans* (79). While *S. aureus* is less susceptible to tigecycline in a mixed biofilm rather than a mono-culture biofilm, there is no effect on *C. albicans* susceptibility to anidulafungin in mixed or mono-culture biofilms (79). Treatment with a combination of anidulafungin and tigecycline improves *S. aureus* killing in a mixed biofilm infection (79). Anidulafungin reduces *S. aureus* poly-β-(1,3)-*N*-acetylglucosamine (PNAG) production, which is a component of *S. aureus* biofilm matrix for select strains (79). Although the mechanism by which anidulafungin reduces PNAG production remains to be determined, the authors suggest that it may inhibit activity of PNAG synthesis protein IcaA (79). However, the anidulafungin-mediated reduction of *C. albicans* biofilm growth may also contribute to enhanced killing of *S. aureus* in biofilm, as reducing *C. albicans* burdens in a polymicrobial biofilm consequently reduces the protective effect of *C. albicans* on *S. aureus* antibiotic resistance (56, 80). One proposed mechanism of reduced antibiotic efficacy towards *S. aureus* in a *C. albicans* biofilm relative to a mono-microbial biofilm is through a physical barrier of the biofilm preventing appropriate diffusion of the antibiotics. To this end, Kong et al. demonstrated that β-glucans present in the ECM of a *C. albicans* biofilm coat the surface of *S. aureus*, preventing penetration of antibiotics (63). However, there may also be a role for chemical signaling in inducing a more antibiotic-tolerant phenotype in *S. aureus* within the polymicrobial biofilm matrix. Exposure of *S. aureus* to physiologic levels of farnesol, the *C. albicans* quorum-sensing molecule that regulates biofilm formation, increases *S. aureus* survival during vancomycin treatment. The decreased vancomycin efficacy may be mediated by a global *S. aureus* stress response following exposure of the bacteria to farnesol (73, 74).

There is an urgent need to develop additional therapeutics to better target microorganisms growing as biofilms. Reducing biofilm biomass, specifically *C. albicans* burdens, improves efficacy of antibiotic treatment (56, 79). Therefore, treatments that aim to reduce biofilm biomass may improve or synergize with additional antimicrobial treatments to improve the outcomes of polymicrobial biofilm infection. Recent work demonstrated that biosurfactants limit formation of staphylococcal and *C. albicans* biofilm growth on plastic and metal surfaces (81, 82). Additionally, extracellular DNA contributes to biofilm biomass, and DNase treatment of *C. albicans* biofilms reduces biomass (83, 84). The use of biosurfactants to prevent biofilm formation and DNase treatment to reduce biofilm biomass may improve antibiotic efficacy against *C. albicans-S. aureus* biofilms.

### Hypoxic Microenvironment in *C. albicans* Biofilm

Sites of infections, particularly invasive infections, are often hypoxic. Hypoxia influences *C. albicans* and *S. aureus* metabolism and subsequent interactions with host cells. Both *C. albicans* and *S. aureus* biofilm growth provide a localized hypoxic environment within the deepest sections of the biofilm (85, 86). Under hypoxic conditions, *C. albicans* induces transcription of genes for iron metabolism, ergosterol and heme biosynthesis, fatty acid metabolism, and cell wall biosynthesis, while reducing transcription of genes involved in mitochondrial respiration and the tricarboxylic acid cycle (87). In addition to regulating *C. albicans* morphogenesis, Efg1p is also a key regulator of biofilm-specific gene expression under hypoxic conditions (88). During invasive *C. albicans* infection, neutrophils are rapidly recruited to the site of infection and contribute to the hypoxic environment (89). In response to hypoxia, *C. albicans* masks β-glucan on its cell surface *via* a mechanism that requires *C. albicans* reactive oxygen species signaling within mitochondria and the cAMP-PKA pathway (90). Increased lactate produced by the recruited neutrophils under hypoxic conditions may also contribute to masking of β-glucan under hypoxic conditions, which leads to enhanced *C. albicans* survival following interactions with neutrophils (89). Hypoxia also impairs neutrophil responses towards *S. aureus*, contributes to *S. aureus* abscess formation, and enhances localized tissue destruction (91, 92). *S. aureus* toxin production is also increased under hypoxic conditions (93). Therefore, it is possible that the hypoxic microenvironment created within a polymicrobial biofilm with *C. albicans* could enhance *S. aureus* toxin production relative to *S. aureus* grown as a mono-culture biofilm (**Figure 2D**). Determining how *C. albicans-S. aureus* biofilm formation alters the distribution or availability of oxygen relative to mono-microbial biofilms will be important to define the consequences of hypoxia on microbial metabolism during polymicrobial biofilm growth.

### *Staphylococcus epidermidis* and *C. albicans* Biofilms

Although typically less severe than the infections caused by *S. aureus, Staphylococcus epidermidis* can also cause recalcitrant biofilm-related infections and can grow as a polymicrobial biofilm with *C. albicans* (23, 94). Early studies determined that *C. albicans* and *S. epidermidis* have greater biomass in the polymicrobial biofilm relative to mono-microbial biofilm growth on a catheter disc model (94). Fluconazole is less effective against *C. albicans* and vancomycin is less effective against some strains of *S. epidermidis* when both organisms are grown in a polymicrobial biofilm (94). *S. epidermidis* added to pre-formed *C. albicans* biofilms adhere to *C. albicans*, and single cell force spectroscopy was used to demonstrate that the strong attachment is primarily mediated by *C. albicans* hyphal proteins Als1 and Als3, as well as *O*-linked mannans on the *C. albicans* cell surface (95, 96). Surgical implantation of a *S. epidermidis-C. albicans* infected catheter in mice resulted in greater *S. epidermidis* growth on the catheter and enhanced dissemination, highlighting a role for *C. albicans* in enhancement of staphylococcal virulence *in vivo* following polymicrobial biofilm growth (83). Microarray analysis revealed 223 differentially expressed *S. epidermidis* genes following biofilm growth with *C. albicans* relative to growth in the absence of *C. albicans* (83). *C. albicans* polymicrobial biofilm growth increases expression of genes encoding the global transcriptional regulator SarA and nucleic acid metabolism pathways, while reducing genes involved in carbohydrate and amino acid metabolism pathways (83). Pammi et al. demonstrated a role for *S. epidermidis* extracellular DNA in contributing to the greater biofilm growth and biomass of a *S. epidermidis-C. albicans* polymicrobial biofilm (83). Taken together, *S. epidermidis*, like *S. aureus*, has enhanced biofilm growth when co-cultured with *C. albicans.* Comparing the metabolic changes that are species-dependent may reveal both shared and unique mechanisms of staphylococcal interactions with fungi during polymicrobial biofilm growth.

## Host-Mediated Mechanisms of Virulence During *C. albicans-S. aureus* Co-Infection

Host immune responses during acute *C. albicans*-*S. aureus* co-infections typically contribute to disease severity rather than promote resolution of infection. While increased virulence during *C. albicans-S. aureus* intraperitoneal co-infection is mediated in part by alterations to microbial physiology, host immune responses also contribute to the lethal synergism of co-infection. Intraperitoneal co-infection of *C. albicans* and *S. aureus* induces increased levels of pro-inflammatory cytokines IL-6 and G-CSF and chemokines CXCL-1, CCL2, and CCL3 as well as greater neutrophil influx to the peritoneal cavity compared to infection with either organism alone (25). The robust inflammatory response during intraperitoneal *C. albicans-S. aureus* infection is driven in part through host inflammatory mediator PGE_2_ (25). Treating mice with a NSAID, which inhibits PGE_2_ production, reduces neutrophil recruitment and improved survival of mice (25). The contribution of *C. albicans*-derived PGE_2_ to lethality during intraperitoneal co-infection of mice is unknown. The addition of pro-inflammatory cytokines also enhances the growth of *S. aureus* in liquid culture (97, 98). Whether the enhanced pro-inflammatory cytokine production during *C. albicans-S. aureus* directly promotes greater *S. aureus* growth during co-infection in the peritoneum is also unknown. Oral co-infection of *C. albicans* and *S. aureus* results in greater dissemination of *S. aureus* to the kidneys relative to *S. aureus* inoculated alone by “hijacking” of macrophages (26, 99–101). It was initially proposed that *S. aureus* binds Als3p on *C. albicans* hyphae and is physically pushed into the subepithelial layer as *C. albicans* hyphae grow, facilitating spread beyond the initial infection site in a “hitchhiking” mechanism (99). However, it was later determined that *C. albicans* hyphae attract macrophages that fail to phagocytose the large hyphal filaments but instead phagocytose *S. aureus* bound to the hyphae in a “bait-and-switch” mechanism (26). The engulfed *S. aureus* evade macrophage-mediated killing, and instead the *S. aureus-*loaded macrophages traffic to the draining lymph node and facilitate bacterial spread to other organs, such as the kidneys (26). Supporting a role of macrophages in enhancing *S. aureus* dissemination during oral co-infection, treatment of mice orally co-infected with *C. albicans* and *S. aureus* with high levels of steroids reduces circulating immune cells and decreases the incidence of *S. aureus* dissemination beyond the oral mucosa to the kidneys (101). There may also be a secondary role for *C. albicans-*induced epithelial damage in mediating *S. aureus* dissemination during oral co-infection. Co-inoculating mice with *S. aureus* and *C. albicans ece1*Δ/Δ, which cannot produce the candidalysin toxin, results in smaller tongue lesions and reduced dissemination to the kidneys relative to co-infection with wild-type *C. albicans* (101). Infection of the oral mucosa with *C. albicans* and *S. aureus* highlights a role for macrophage phagocytosis in facilitating dissemination of *S. aureus* beyond the initial site of infection.

*C. albicans* and *S. aureus* secreted factors produced during co-infection also negatively impact host cells by direct toxicity. Epithelial cell lines exhibit greater cell death (as measured by lactate dehydrogenase release) following treatment with conditioned media from *C. albicans-S. aureus* biofilms compared to treatment with conditioned media from either organism grown as a mono-microbial biofilm (102) . *S. aureus* mono-culture biofilm growth produces higher levels of toxins leukocidin A/B and α-toxin compared to planktonic cell growth, which inhibits macrophage phagocytosis and induces macrophage cytotoxicity (103). Because *C. albicans* enhances *S. aureus* α-toxin secretion in planktonic co-culture, *C. albicans* may further enhance *S. aureus* toxin production in a polymicrobial biofilm (28). Chemical signals produced by *C. albicans-S. aureus* biofilms also target host cells (104). Farnesol, the *C. albicans* quorum sensing molecule that impacts both *C. albicans* and *S. aureus* biofilm growth, induces apoptosis of human squamous carcinoma cells (105). Additionally, even brief exposure of pre-osteoblastic cell lines to farnesol inhibited cell spreading, suggesting that chemical signaling from polymicrobial biofilms that form on orthopedic devices may influence bone cell physiology (106). Additional work to understand the full effects of polymicrobial biofilm growth on host cell responses during infection is needed. Because host responses can be influenced by the local environment of the infection site, it will be important to analyze host responses during *C. albicans-S. aureus* co-infection in multiple models.

## *C. albicans* and *S. aureus* Influence on Secondary Infections

There is significant overlap in immune responses to both *C. albicans* and *S. aureus*. For example, a Th1/Th17 response is required for clearance of both *S. aureus* and *C. albicans* in mice (107). STAT3 is activated downstream of Th17 cytokine signaling and is important for clearance of *C. albicans* and *S. aureus.* STAT3 deficiencies predispose individuals to both *S. aureus* and *C. albicans* skin and mucosal infections (108). IL-17, a Th17 signature cytokine, is important for host defense against both *C. albicans* and *S. aureus* skin and mucosal infections (109). IL-17 induces epithelial cells to produce antimicrobial peptides as well as CXC chemokines, which contribute to neutrophil recruitment (109). Pro-inflammatory cytokines, antimicrobial peptides, and neutrophils are important for *S. aureus* clearance during skin infection, while the epithelial cell-derived antimicrobial peptides are critical for protection against *C. albicans* mucosal infections (110–112). Additionally, a vaccine derived from the *C. albicans* Als3 protein not only provides protection against *C. albicans* infections but also *S. aureus* bacteremia and skin and soft tissue infection, and the protection against both organisms is mediated *via* STAT3 and Th17 signaling (113–115). The dual-species protection of the Als3p-derived vaccine may result from structural similarities between Als3p and *S. aureus* surface adhesins, such as collagen binding protein and clumping factor (116). Interestingly, Als3p is also the main factor to which *S. aureus* binds on *C. albicans* hyphal filaments, although this interaction was not shown to involve *S. aureus* collagen binding protein or clumping factor (59, 99).

**Figure 3:**
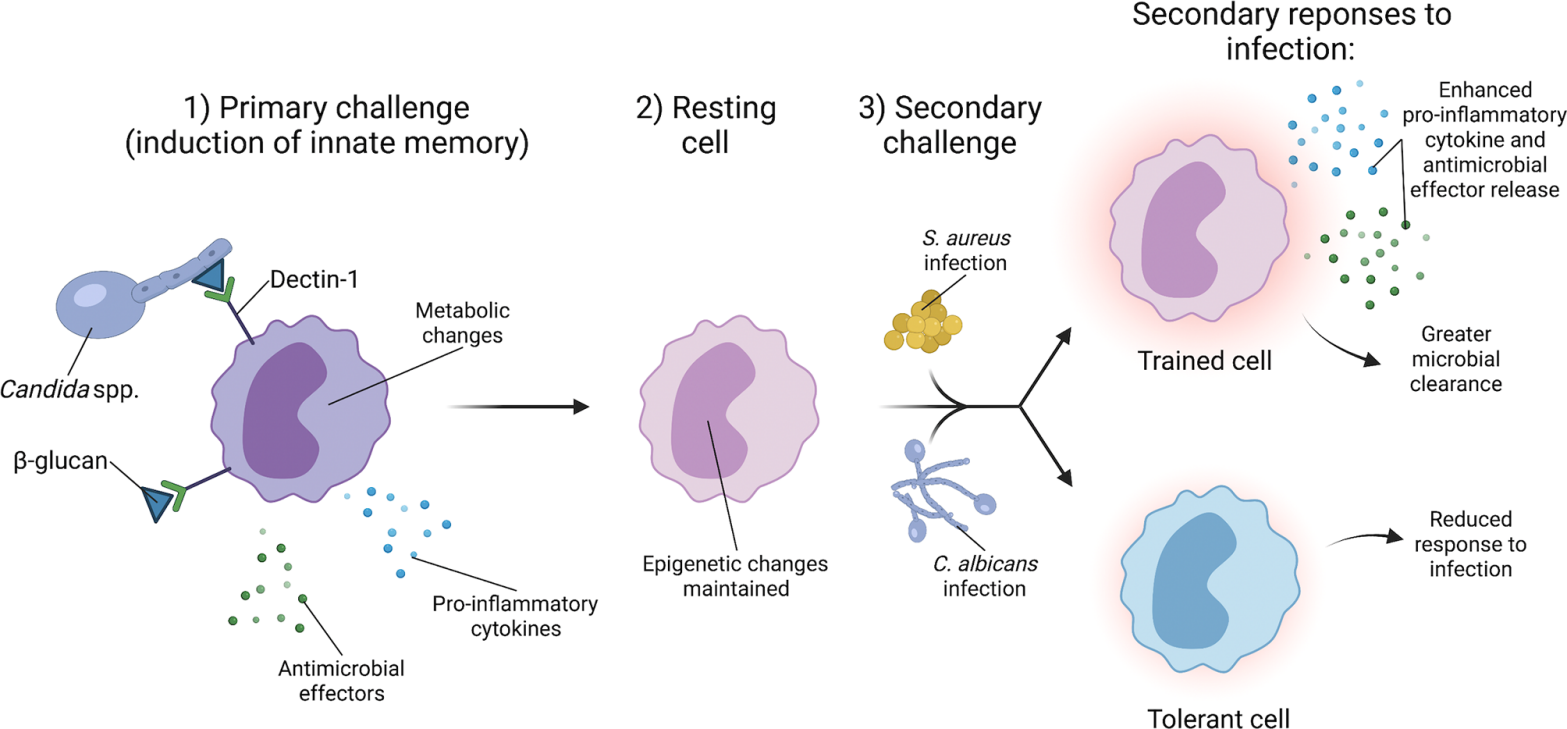
*C. albicans* induces innate immune training to provide broad protection against secondary infections. The generation of innate immune memory is dependent upon *C. albicans* cell wall component β-glucan signaling through Dectin-1 on monocytes. Dectin-1 signaling induces epigenetic changes and a metabolic shift from oxidative phosphorylation to aerobic glycolysis. Upon clearance of the primary *C. albicans* infection, monocytes enter a resting state, but retain the epigenetic changes from the primary challenge. Secondary challenge with a lethal infection of *C. albicans* or other organisms such as *S. aureus* causes the trained monocyte to produce a robust immune response that facilitates greater clearance of secondary *S. aureus* and *C. albicans* infections.

In addition to providing cross-protection *via* adaptive immune responses, *C. albicans* also induces protection against secondary *S. aureus* infections through innate immune memory, often referred to as trained immunity (**Figure 3**). Trained immunity is the process by which an initial infection induces epigenetic changes in innate immune cells that alter their response to a secondary infection (117, 118). A key feature of trained immunity is that the immune cell returns to a baseline after the initial infection is cleared, but the epigenetic changes induced by the initial infection persist (119). During a secondary infection, the trained immune cells generate a robust immune response that contributes to clearance of the secondary infection (119). *C. albicans* infection and the fungal cell wall protein β-glucan are potent inducers of trained immunity. Non-lethal *C. albicans* injection in mice provides protection against a secondary lethal *C. albicans* challenge (27, 120). Protection occurs *via C. albicans* β-glucan signaling through Dectin-1 on monocytes, which is associated with changes in histone methylation, indicating an epigenetic mechanism (120). Powering the trained immunity effect induced by β-glucan is a shift in monocyte metabolism from oxidative phosphorylation to aerobic glycolysis (121). Additionally, training induced by *C. albicans* β-glucan not only provides protection against a secondary *C. albicans* infection, but also secondary challenge by other microorganisms, including *S. aureus* (27, 121, 122). These data indicate that host exposure to *C. albicans* influences subsequent *S. aureus* infection by providing protection *via* innate immune memory. However, the protection generated during primary infection may depend on the degree of immune stimulation by *C. albicans*. A high infectious dose of *C. albicans* induces a tolerant phenotype in monocytes *in vitro*, a state in which monocytes have a diminished response to secondary challenge (118, 123). The amount of β-glucan exposure during infection may also vary based on the infection site and local environment. Typically, β-glucan comprises the inner cell wall layer of *C. albicans*, and *C. albicans* can modulate levels of β-glucan display in the cell wall during infection in response to host cues such as pH or available lactate (67, 124–126). Therefore, *C. albicans* regulation of β-glucan exposure *in vivo* likely influences the training phenotype. Other signaling mechanisms stimulated during infection may also contribute to the generation of innate immune cell memory. Treatment of mice with monophosphoryl lipid A, a Toll-like receptor 4 agonist, induces protection against a secondary infection with either *C. albicans* or *S. aureus* (127, 128). Cell signaling pathways induced by multiple PAMPs during infection, particularly polymicrobial infections, may influence the development of innate immune memory.

Innate immune training by *Candida* also influences the outcome of polymicrobial infection. Lilly et al. revealed that inoculation of mice intraperitoneally with other *Candida* species, such as *C. dubliniensis*, provides protection against a secondary co-infection challenge with a lethal dose of *C. albicans* and *S. aureus* (129). Mice that are rechallenged have greater neutrophil recruitment to the peritoneal cavity as well as enhanced *C. albicans* clearance, suggesting a role for neutrophils in providing protection against secondary infection (129). Inoculation of mice with *C. dubliniensis* provides protection against a lethal *C. albicans-S. aureus* infection for up to 60 days and was not dependent upon B and T cells (130). One hypothesis for *C. dubliniensis*-mediated immune protection is that following inoculation of certain *Candida* species, the bone marrow is transiently colonized, myelopoiesis is altered to skew cell development towards suppressor cells, and upon secondary challenge by a lethal polymicrobial infection, suppressive cells are activated that minimize the harmful pro-inflammatory response that occurs when naïve mice are co-infected (130, 131). Whether the protective effect conferred by primary *C. dubliniensis* inoculation is also mediated by β-glucan and Dectin-1 signaling is unknown.

While *Candida* and β-glucan induce a robust trained immunity response, *S. aureus* has a more limited ability to induce innate immune memory. Inoculating mice with *S. aureus* to induce a skin and soft tissue infection (SSTI) conferred protection against a secondary challenge with *S. aureus* after the initial infection cleared (132). Macrophages isolated from the *S. aureus-*infected mice were able to induce greater killing of *S. aureus* when infected *ex vivo* as compared to macrophages isolated from naïve mice (133). Enhanced macrophage-mediated killing is specific for *S. aureus* as the macrophages from *S. aureus-*infected animals did not enhance killing of *S. epidermidis, Enterococcus faecalis*, or *E. coli* (133). Additionally, protection against secondary SSTI infection is limited to the site of inoculation and is dependent upon the resident dermal macrophages present at the site of inoculation (134). Transfer of bone marrow cells from mice that received an initial *S. aureus* intradermal challenge failed to induce protection against secondary challenge. This differs from training with β-glucan, which induces changes in the hematopoietic progenitor cells of the bone marrow (134, 135). *S. aureus* may also negatively impact trained immunity during growth as small colony variants, or SCVs. SCVs can arise from mutations in metabolic genes and are associated with chronic *S. aureus* infections due to their long-term persistence within host cells and increased resistance to standard treatments (136). *S. aureus* Δ*hemB* is a SCV with altered metabolic activity that fails to induce protection against a secondary *S. aureus* challenge in a murine intradermal infection model (137). The absence of trained immunity was proposed to be the result of an enhanced use of fumarate by *S. aureus* Δ*hemB*, which depletes this metabolite from the local environment and prevents macrophages from using fumarate to drive the epigenetic changes required for training (137). Taken together, these studies demonstrate that *S. aureus* can produce a localized trained immunity that protects against secondary *S. aureus* challenge in an SSTI murine model of infection, and the protection is in part due to host metabolism as well as the activity of resident dermal macrophages.

## Conclusions and Future Perspectives

*C. albicans* and *S. aureus* are formidable opportunistic pathogens, and their interactions during acute polymicrobial infection further worsen disease. Physical interactions mediate tight association of *S. aureus* with *C. albicans* hyphal filaments, facilitating enhanced chemical signaling and metabolite exchange between the two organisms. Adherence of *S. aureus* to *C. albicans* is particularly important for polymicrobial biofilm growth, which reduces antibiotic efficacy due to physical shielding of *S. aureus* from antibiotics as well as influencing *S. aureus* stress responses. *C. albicans-S. aureus* co-infection induces a severe pro-inflammatory response characterized by neutrophil influx, greater microbial burdens, and cytokine responses. Paradoxically, separating the timing of *C. albicans* and *S. aureus* inoculation induces a protective effect due to the induction of trained immunity by *C. albicans* that provides protection against a secondary *S. aureus* infection. Metabolic changes during co-infection underpin a variety of outcomes of *C. albicans-S. aureus* interactions, particularly during biofilm growth and when interacting with host cells. It is important to establish clinically relevant models of polymicrobial infection, particularly chronic infection models, to better characterize metabolic changes during *C. albicans-S. aureus* co-infection. Future studies that examine molecular interactions among *C. albicans, S. aureus*, and host immune cells during polymicrobial infection are needed to identify therapies that may target these challenging infections. Determining how *C. albicans* and *S. aureus* physiology changes during various co-infection models may reveal additional mechanisms of interaction that can be targeted to reduce mortality and enhance antimicrobial efficacy.

## Author Contributions

KE drafted the manuscript, which was edited by KE and JC. All authors contributed to the article and approved the submitted version.

## Funding

JC was supported by R01AI132560 (NIAID), R01AI145992 (NIAID), R01AI161022 (NIAID), a Senior Research Award from the Crohn’s and Colitis Foundation, and a Career Award for Medical Scientists from the Burroughs Wellcome Fund. KE was supported by 2T32AI095202-12.

## Conflict of Interest

The authors declare that the research was conducted in the absence of any commercial or financial relationships that could be construed as a potential conflict of interest.

## Publisher’s Note

All claims expressed in this article are solely those of the authors and do not necessarily represent those of their affiliated organizations, or those of the publisher, the editors and the reviewers. Any product that may be evaluated in this article, or claim that may be made by its manufacturer, is not guaranteed or endorsed by the publisher.
